# The contribution of mutations in *MYH7 *to the onset of cardiomyopathy

**DOI:** 10.1007/s12471-017-1045-5

**Published:** 2017-10-20

**Authors:** I. A. E. Bollen, J. van der Velden

**Affiliations:** Physiology, Amsterdam Cardiovascular Sciences, VU University Medical Center, Amsterdam, The Netherlands

In this issue of the Netherlands Heart Journal, van der Linde et al. describe a novel Dutch founder mutation (*MYH7*_*p.Asn1918Lys*_ *)* in *MYH7*, the gene encoding myosin heavy chain 7, leading to cardiomyopathy and congenital heart defects [1].

Van der Linde et al. showed that the *MYH7*_*p.Asn1918Lys*_ mutation resulted in predominantly dilated cardiomyopathy (DCM) but was also present in patients suffering from peripartum cardiomyopathy (PPCM), non-compaction cardiomyopathy (NCCM) and congenital heart defects [1]. Mutations in many different genes have been implicated to be causal to DCM [2]. However, the same genes are often also implicated in the onset of other cardiomyopathies such as hypertrophic cardiomyopathy (HCM), NCCM or restrictive cardiomyopathy [2]. It is therefore difficult to understand how these mutations contribute to the onset of disease and why one gene mutation causes diverse cardiomyopathy phenotypes. This is especially the case for mutations in *MYH7* which has been suggested to be an important gene in HCM as well as DCM pathogenesis.

Gene variants can be benign and it is often difficult to establish which variants are pathogenic. The Exome Aggregation Consortium (ExAC) has recently re-assessed various mutations assumed to be causal to the onset of cardiomyopathy [3]. According to the ExAC, many variants previously assumed to be causal in DCM such as mutations in *MYH6, SCN5A* and *MYBPC3* were not found in excess in the DCM population compared with ~60,000 controls, making it unlikely that they played a role in theonset of DCM [3]. *MYH7* variants were greatly enriched in the HCM population compared with the ExAC controls with rare variants in *MYH7* being accountable for 14.2% of the variation observed in HCM [3]. The prevalence of variants in the *MYH7* gene was slightly higher (5.3%) in the DCM population compared with ExAC controls which implies that variants found in *MYH7* are possible disease-causing mutations in DCM [3]. The *MYH7*_*p.Asn1918Lys*_ mutation described by van der Linde et al. was absent in ExAC controls [1]. The *MYH7*_*p.Asn1918Lys*_ mutation seems to be more associated with DCM than HCM as shown by van der Linde et al. [1].

One factor that is important in the pathogenic effects of mutations is the location of the mutation. For example, mutations causal to HCM are enriched in the part of the *MYH7* gene encoding the myosin head domain, while *MYH7* mutations related to DCM are more disperse across the entire *MYH7* gene [3]. The *MYH7*_*p.Asn1918Lys*_ mutation is located in exon 39, the C‑terminus of the protein encoding the tail domain. Pathogenic effects of mutations located in the globular head domain affect cross-bridge kinetics of contraction and energy consumption [4, 5] and stiffness of myosin heads [6]. The *MYH7*_*p.Asn1918Lys*_ mutation is likely to exert its pathogenic effect in another manner. Considering the location of the mutation in the coiled-coil rod region, the most likely pathogenic affects would be impairment of the incorporation of myosin into the myofilaments or binding to titin [7]. However, functional studies are needed in order to confirm the true pathogenic effect of this mutation.

Van der Linde et al. describe a relatively benign course in patients carrying the *MYH7*_*p.Asn1918Lys*_ mutation [1]. This makes it likely that the pathogenic effect of the mutation is also mild. Therefore, it might be possible that the *MYH7*_*p.Asn1918Lys*_ mutation acts as a disease modifier in which cellular dysfunction is aggravated leading to onset of disease, an earlier onset of disease, or progression of the disease. This could also explain why the mutation was found in DCM, PPCM, NCCM and congenital heart disease patients and why some patients were very young. If this is the case, a second disease hit is likely to be present contributing to the onset of disease. The study by van der Linde et al. showed a disease penetrance of 47.7% among mutation carriers [1], which implies the mutation, like many other reported mutations, is not the sole cause of disease.

Studies assessing the prevalence of mutations in large cohorts such as the current study give important insight into the prevalence of genetic variants, the likelihood of pathogenicity, and their effects on disease presentation. This information is needed to improve risk assessment in mutation carriers and patient counseling.

## References

[1] van der Linde IHM, Hiemstra YL, Bokenkamp R (2017). A Dutch MYH7 founder mutation, p.(Asn1918Lys), is associated with early onset cardiomyopathy and congenital heart defects. Neth Heart J.

[2] Hershberger RE, Hedges DJ, Morales A (2013). Dilated cardiomyopathy: the complexity of a diverse genetic architecture. Nat Rev Cardiol.

[3] Walsh R, Thomson KL, Ware JS (2017). Reassessment of Mendelian gene pathogenicity using 7,855 cardiomyopathy cases and 60,706 reference samples. Genet Med.

[4] Witjas-Paalberends ER, Ferrara C, Scellini B (2014). Faster cross-bridge detachment and increased tension cost in human hypertrophic cardiomyopathy with the R403Q MYH7 mutation. J Physiol.

[5] Bloemink M, Deacon J, Langer S (2014). The hypertrophic cardiomyopathy myosin mutation R453C alters ATP binding and hydrolysis of human cardiac beta-myosin. J Biol Chem.

[6] Seebohm B, Matinmehr F, Kohler J (2009). Cardiomyopathy mutations reveal variable region of myosin converter as major element of cross-bridge compliance. Biophys J.

[7] Colegrave M, Peckham M (2014). Structural implications of beta-cardiac myosin heavy chain mutations in human disease. Anat Rec (Hoboken).

